# Antibiotic Stewardship Program at a Tertiary Care Academic Hospital: A Comprehensive Analysis of an Audit Across Various Specialties

**DOI:** 10.7759/cureus.107504

**Published:** 2026-04-21

**Authors:** Arpana Singh, Garima Mittal, Rajender Singh, Arushi Gupta, Sohaib Ahmad, Ravinder S Saini

**Affiliations:** 1 Microbiology, Himalayan Institute of Medical Science, Swami Rama Himalayan University, Dehradun, IND; 2 Internal Medicine, Himalayan Institute of Medical Science, Swami Rama Himalayan University, Dehradun, IND; 3 Hospital Management, Himalayan Institute of Medical Science, Swami Rama Himalayan University, Dehradun, IND

**Keywords:** antimicrobial resistance(amr), antimicrobial stewardship, guideline adherence, hospital audit, who aware classification

## Abstract

Introduction

Antimicrobial resistance (AMR) is a growing global health concern, largely driven by inappropriate antibiotic use in hospital settings. Antimicrobial stewardship programs (AMSPs) are essential to promote rational antibiotic prescribing; however, variability in implementation and adherence across specialties remains a challenge. Periodic audits are critical for assessing prescribing practices and identifying areas for improvement. This study was conducted to evaluate the implementation of an AMSP at a tertiary care academic hospital through a cross-specialty audit of antibiotic use.

Methods

A cross-sectional audit of 800 patient records from January to December 2023 was conducted across multiple specialties. Antibiotic prescribing patterns, adherence to institutional guidelines, and classification according to the WHO Access, Watch, and Reserve (AWaRe) framework were analyzed. Comparisons were made between medical and surgical specialties.

Results

The overall antibiotic prescription rate was 88.1% (705/800 cases), with significant variation across departments. Ophthalmology demonstrated 100% adherence to institutional guidelines (100/100 cases), whereas Medicine and Pediatrics showed comparatively lower adherence (45/100 (45%) and 40/100 (40%), respectively). De-escalation practices were suboptimal, particularly in critical care settings. Watch group antibiotics predominated across both specialties, and a statistically significant difference in AWaRe distribution between medical and surgical departments was observed (p = 0.003). Surgical departments exhibited significantly higher adherence to institutional antibiotic guidelines compared with medical departments (330/400 (82.5%) vs 278/400 (69.6%), p < 0.001). They also prescribed fewer antibiotics per patient (mean 1.18 ± 0.5 vs 1.43 ± 0.6, p < 0.001) and had shorter durations of antibiotic therapy (3.91 ± 1.1 vs 4.25 ± 1.2 days, p < 0.001).

Conclusion

AMSP implementation has led to improvements in antibiotic prescribing practices; however, important specialty-specific gaps persist, particularly in medical and critical care settings. The high use of Watch group antibiotics and limited de-escalation highlight the need for strengthened stewardship efforts. Targeted interventions, regular audit and feedback, and the development of specialty-specific guidelines are essential to optimize antibiotic use and sustain the impact of stewardship programs.

## Introduction

Resistance to antimicrobials poses a serious threat to global health and has been largely attributed to the improper use of antibiotics [1]. The WHO estimates that antimicrobial resistance (AMR) will lead to approximately 10 million deaths by 2050 [2,3]. Hospital antibiotic policies are essential for promoting rational antimicrobial use; however, their effectiveness largely depends on adherence by healthcare providers [4].

Resistance to carbapenems has been noted to be quite high and ranges between 41% and 46%. Similar trends have been noted with other widely used antibiotics, such as amikacin (44.8%), piperacillin-tazobactam (57.9%), co-trimoxazole (63.1%), cefepime (69.1%), ciprofloxacin (62.1%), and cefotaxime (79.5%), as suggested by the annual report of the National Antimicrobial Resistance Surveillance Network (NARS-Net India 2021) [5]. These findings underscore the urgent need for optimized antibiotic use.

Inappropriate prescribing practices, such as the unnecessary use of broad-spectrum antibiotics, incorrect dosing, prolongation of therapy beyond what is required, and prolonged hospital stays, can contribute significantly to the development of AMR. These practices also add to healthcare costs and increase the likelihood of adverse drug events [6]. It has been reported that nearly half of antibiotic prescriptions in hospital settings may be inappropriate or avoidable [7,8], underscoring the need for regular monitoring through structured audits.

Hospital antibiotic stewardship policies strive to provide guidance on antimicrobial choice, dose, and duration based on available evidence [9]. Yet, the existence of such guidelines does not always equate to their implementation [10]. Routine assessment of prescribing patterns is, therefore, critical not only to examine the extent of concordance with guidelines but also to identify areas requiring improvement and to inform targeted antimicrobial stewardship initiatives [11]. In this respect, the WHO AWaRe classification serves as a useful framework for the assessment of prescribing patterns, ideally with a target of 60% or more of antibiotic consumption from the “Access” group [12].

Although an Antibiotic Stewardship Program has been in place since 2017 with the aim of encouraging rational antibiotic use, there is still limited information on how well hospital antibiotic policies are being followed in the sub-Himalayan region [13]. Keeping this gap in mind, the present study was undertaken to assess antibiotic prescribing practices through a detailed audit in a tertiary care hospital. Such audits not only help improve prescribing practices at the institutional level but also contribute to broader epidemiological control of AMR by limiting the emergence and spread of resistant organisms.

## Materials and methods

This study was conducted at the Himalayan Institute of Medical Science (HIMS), Dehradun, between January and December 2023 after obtaining clearance from the Institutional Ethics Committee. The study evaluated antibiotic prescribing practices and adherence to institutional guidelines across different medical and surgical departments. Patients admitted to the departments of General Medicine, Critical Care Medicine (CCM), Paediatrics, Pulmonary Medicine, Ophthalmology, Obstetrics and Gynaecology, General Surgery, and Urology were included in the study. A total of 800 admitted patients (100 patients from each of the eight specialties) were selected using systematic random sampling from departmental medical records over the study period. This observational study was conducted and reported in accordance with the Strengthening the Reporting of Observational Studies in Epidemiology (STROBE) guidelines.

Inclusion criteria

All adult inpatients admitted during the study period who received at least one systemic antibiotic and had a hospital stay of ≥24 hours, with complete clinical and prescription records available for analysis.

Exclusion criteria

Outpatients, patients with a hospital stay of less than 24 hours, those not receiving antibiotics, cases with incomplete records, and repeat admissions of the same patient were excluded.

Data were collected using a standardized antibiotic audit form designed in accordance with the hospital’s antibiotic policy guidelines. The parameters evaluated included the clinical diagnosis and details of the antibiotics prescribed, including the name of the antibiotic, appropriate dose, route of administration, frequency, and duration of therapy. In addition, documentation of the indication for antibiotic use, adherence to the institutional antibiotic policy, total duration of therapy, and length of hospital stay were also assessed.

The audit was conducted by members of the Hospital Infection Prevention and Control (HIPC) team, who reviewed randomly selected patient medical records. Each prescription was assessed for appropriateness based on the hospital's antibiotic policy guidelines. Compliance with the hospital antibiotic policy was evaluated based on the appropriate choice and duration of antibiotic for the indication, correct dosing and frequency, review and revision of antibiotics, and adherence to de-escalation protocols.

Antibiotics were further categorized according to the WHO 2019 AWaRe classification [14]. The AWaRe classification groups antibiotics into three categories: Access (first- and second-line antibiotics with lower resistance potential), Watch (antibiotics with higher resistance potential and recommended as first- or second-choice only for specific indications), and Reserve (last-resort antibiotics used for multidrug-resistant infections). De-escalation rate was defined as the proportion of patients in whom initial broad-spectrum antibiotic therapy was either switched to a narrower-spectrum agent or discontinued based on clinical improvement and/or microbiological results.

Statistical analysis 

Data were entered into Microsoft Excel and analyzed using GraphPad Prism 9.1.2 (226) software (San Diego, CA, USA). Frequencies and percentages were used to express categorical variables, while mean ± standard deviation was used to summarize continuous variables. The chi-square test was used for comparison of categorical variables, while Student’s t-test was used for comparison of continuous variables, wherever applicable. A p-value of < 0.05 was considered statistically significant.

## Results

Data were analysed from 800 patients admitted across eight departments: General Medicine, Pulmonary Medicine, Critical Care Medicine (CCM), Paediatrics, General Surgery, Obstetrics and Gynaecology, Urology, and Ophthalmology during the study period.

**Figure 1:**
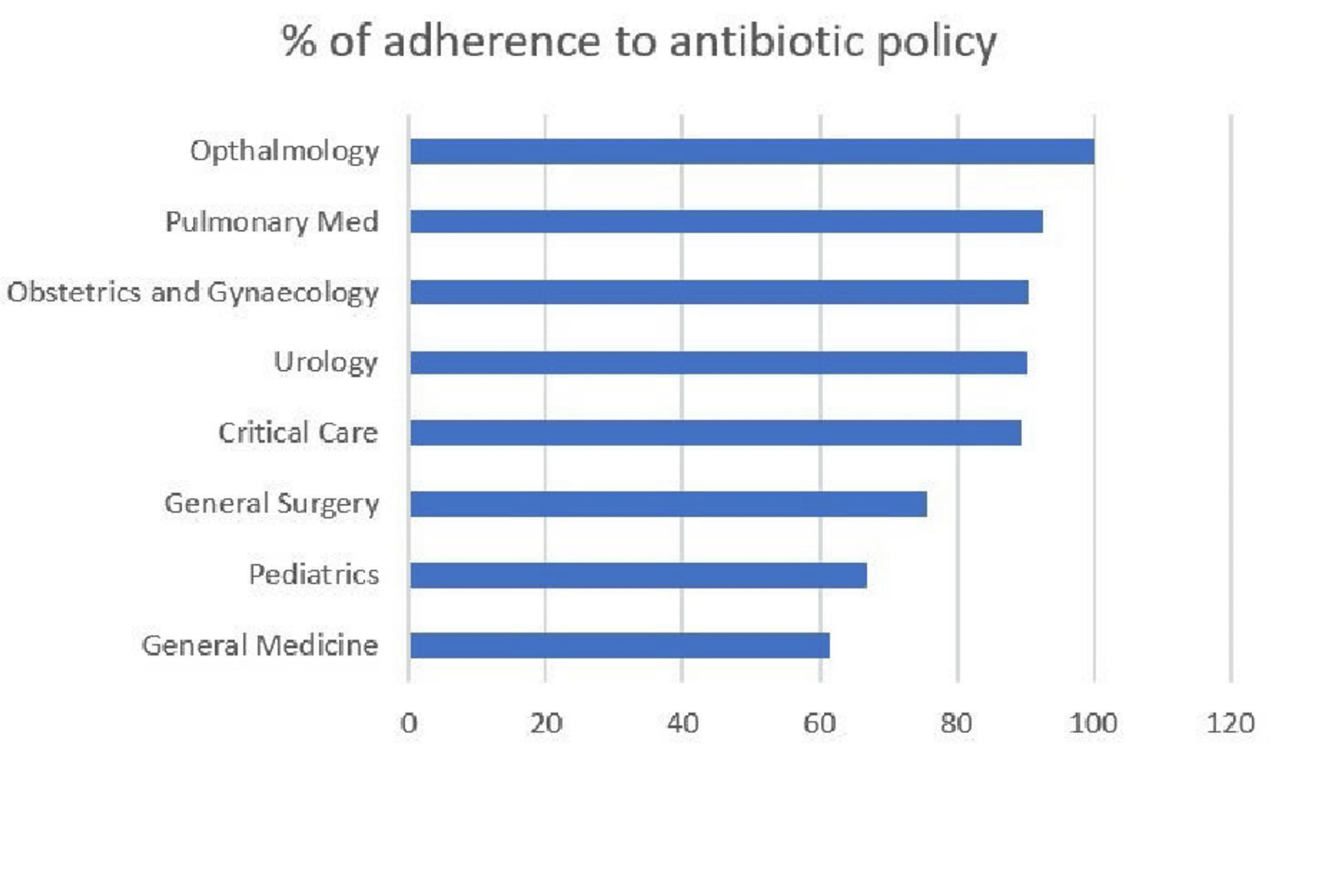
Adherence to institutional antibiotic prescribing guidelines (%). A total of N = 800 patients were included in the study, with n = 100 patients from each department (General Medicine, Paediatrics, General Surgery, Critical Care Medicine, Urology, Obstetrics and Gynaecology, Pulmonary Medicine, and Ophthalmology). Percentages represent the proportion of cases with antibiotic prescriptions compliant with hospital policy (denominator = 100 per department).

**Figure 2:**
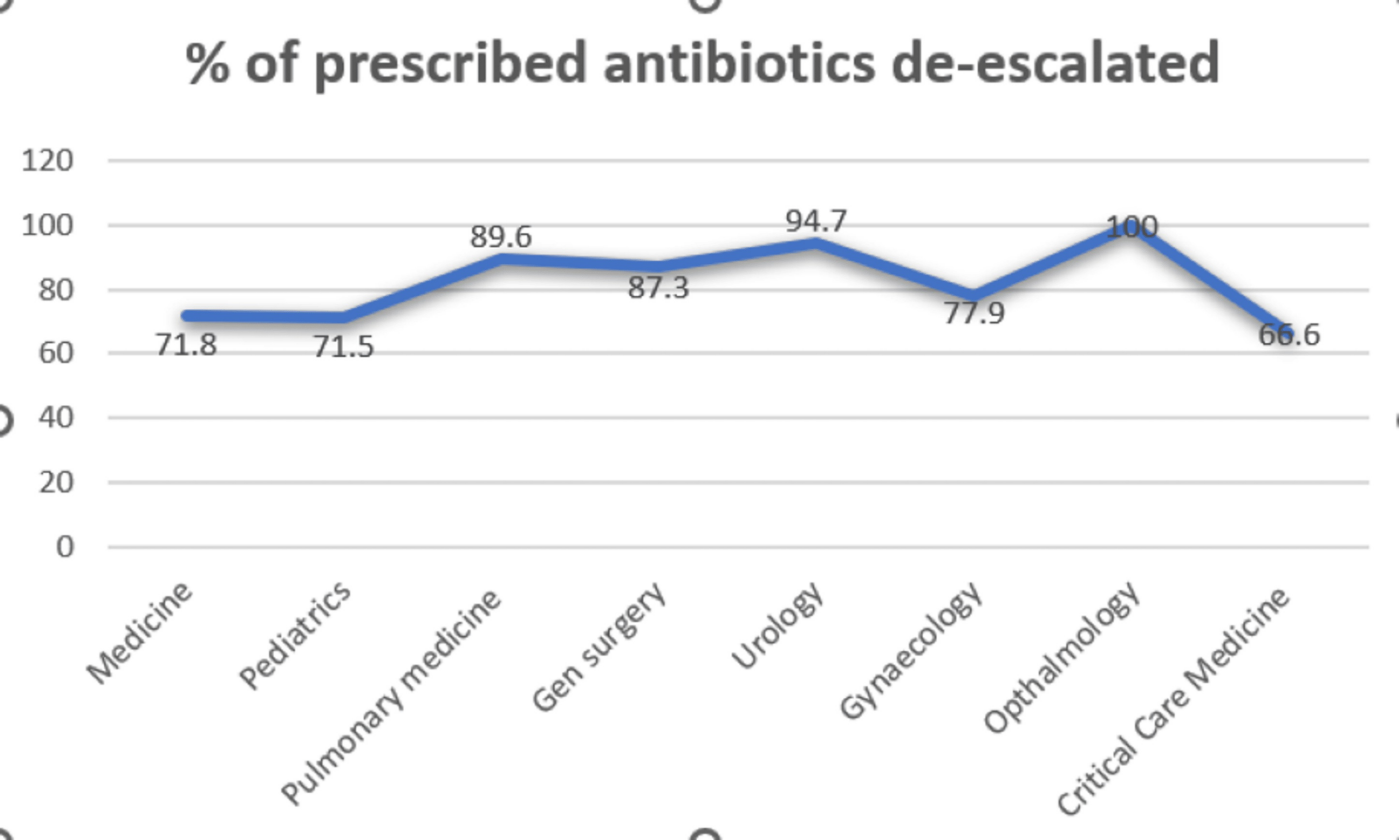
Percentage of antibiotic prescriptions undergoing de-escalation. A total of N = 800 patients were included in the study, with n = 100 patients from each department (General Medicine, Critical Care Medicine, Paediatrics, Pulmonary Medicine, General Surgery, Urology, Obstetrics and Gynaecology, and Ophthalmology). The percentages represent the proportion of cases in which antibiotic therapy was de-escalated out of the total number of patients receiving antibiotics in each department (denominator = patients receiving antibiotics in the respective department).

Significant interdepartmental variation was observed in adherence to hospital antibiotic policy and de-escalation practices. Ophthalmology demonstrated 100% adherence along with a high de-escalation rate (95%). Obstetrics and Gynaecology also showed high adherence (90%) with a de-escalation rate of 80%. Pulmonary Medicine and CCM reported adherence rates of 85% and 80%, respectively, with corresponding de-escalation rates of 89.6% and 66.6%. General Surgery and Urology showed moderate adherence (70% and 75%, respectively) with relatively higher de-escalation rates (87% and 94%). In contrast, General Medicine and Paediatrics reported lower adherence (45% and 40%, respectively) and comparatively lower de-escalation rates (~71%) (Figures 1-2).

**Figure 3:**
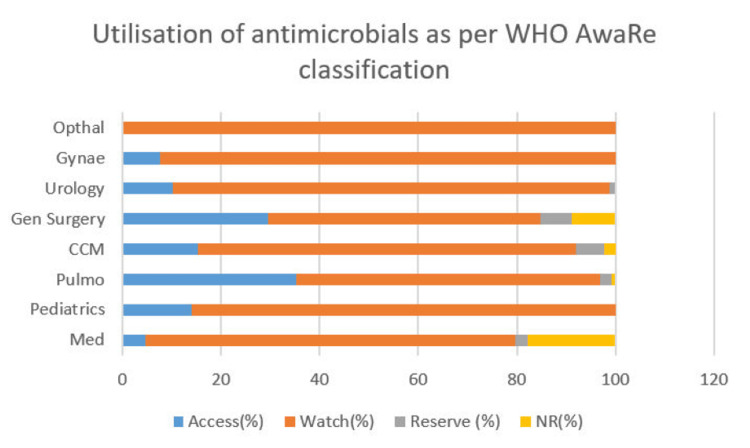
Utilization pattern of antimicrobials based on WHO AWaRe categories. A total of 871 antibiotics were prescribed across eight departments, with the number of antibiotics in each department as follows: Medicine (n = 108), Paediatrics (n = 71), Pulmonary Medicine (n = 133), Critical Care Medicine (n = 137), General Surgery (n = 112), Urology (n = 106), Obstetrics and Gynaecology (n = 104), and Ophthalmology (n = 100). Percentages represent the proportion of antibiotics in each AWaRe category (Access, Watch, Reserve, and Not Recommended) within each department (denominator = total antibiotics prescribed in that department). AWaRe: Access, Watch, Reserve; CCM: Critical Care Medicine.

Analysis of antimicrobial utilization based on the WHO AWaRe classification revealed a predominance of ‘Watch’ group antibiotics across all departments. Ophthalmology reported nearly exclusive use of Watch antibiotics, whereas CCM demonstrated a more balanced distribution, including approximately 20% Access antibiotics and a small proportion of Reserve antibiotics. Similar patterns were observed across other departments (Figure 3).

A statistically significant difference in AWaRe distribution was observed between medical and surgical specialties (χ² = 13.83, p = 0.003). Surgical specialties showed a higher proportion of Watch group antibiotic use, whereas Access group usage was relatively higher in medical specialties (Table 1).

**Table 1:** Distribution of antibiotics according to the WHO AWaRe classification in medical and surgical departments. Values are expressed as number (%). The total number of antibiotics prescribed was 449 in medical departments and 422 in surgical departments. Medical departments included General Medicine, Critical Care Medicine, Pulmonary Medicine, and Paediatrics, while surgical departments included General Surgery, Urology, Obstetrics and Gynaecology, and Ophthalmology. The number of patients in the medical and surgical departments was 400 each. A statistically significant difference in AWaRe distribution between the two groups was observed (χ² = 13.83, p = 0.003). AWaRe: Access, Watch, and Reserve.

AWaRe category	Medical departments, n (%)	Surgical departments, n (%)
Access	83 (18.5)	52 (12.3)
Watch	329 (73.3)	352 (83.4)
Reserve	14 (3.1)	8 (1.9)
Not recommended	23 (5.1)	10 (2.4)

Comparison between medical and surgical departments revealed significant differences across multiple parameters (Table 2). Prescriptions adhering to standard guidelines were significantly more frequent in surgical departments (330/400, 82.5%) than in medical departments (278/400, 69.6%) (χ² = 18.53, p < 0.001). The mean duration of antibiotic use was slightly longer in medical departments (4.25 ± 1.2 days) than in surgical departments (3.91 ± 1.1 days), and this difference was statistically significant (t = 4.18, p < 0.001). Similarly, the average number of antibiotics prescribed per patient was higher in medical departments (1.43 ± 0.6) than in surgical departments (1.18 ± 0.5) (t = 6.39, p < 0.001). A significant difference was also observed in the duration of hospitalization, with a greater proportion of patients in medical departments having shorter hospital stays of ≤7 days (58.0% vs 21.3%), whereas prolonged hospitalization (>7 days) was more common in surgical departments (78.7% vs 42.0%).

**Table 2:** Comparison of antibiotic prescribing practices and clinical parameters between medical and surgical departments. Categorical variables are expressed as number (percentage), and continuous variables as mean ± SD. Percentages were calculated using the total number of patients in each group (n = 400 for medical departments and n = 400 for surgical departments). The chi-square test was used for comparison of categorical variables, and the independent-samples t-test was used for continuous variables. *Indicates a statistically significant difference (p < 0.05).

Parameters	Medical Departments (n = 400)	Surgical Departments (n = 400)	Test statistic	p-value
Prescriptions following guidelines, n (%)	278 (69.5)	330 (82.5)	χ² = 18.53	<0.001*
Duration of antibiotic usage (days), mean ± SD	4.25 ± 1.2	3.91 ± 1.1	t = 4.18	<0.001*
Number of antibiotics per patient, mean ± SD	1.43 ± 0.6	1.18 ± 0.5	t = 6.39	<0.001*
Duration of hospitalisation, n (%)				
≤7 days	232 (58.0)	85 (21.3)	χ² = 112.92	<0.001*
>7 days	168 (42.0)	315 (78.8)		

## Discussion

To the best of our knowledge, this is among the first antibiotic audits from the sub-Himalayan region, and published data on prescribing practices from this region are quite limited. This gap in evidence, especially from a geographically diverse setting, prompted us to undertake the present study. In our cohort, the rate of antibiotic prescribing was high, at 88.1% of cases (705/800), suggesting high overall antimicrobial use. This figure is slightly higher than that reported by Pauwels I et al. (79.4%) [15,16].

Antibiotics from the “Watch” group were the most commonly used across departments when antimicrobial utilization was evaluated according to the WHO AWaRe classification framework. This pattern is alarming, since these agents carry a higher risk of promoting the emergence of resistance. Similar prescribing trends have been reported in other middle-income settings, where clinicians are often guided by spectrum of coverage, local resistance patterns, access considerations, and disease burden [15-18].

Although medical specialties used a relatively higher proportion of Access antibiotics compared with surgical units, the overall dependence on Watch antibiotics remained evident.

We also observed adherence to the institutional antibiotic policy across different departments while prescribing antibiotics. Medical departments showed lower adherence than surgical departments (69.6% vs 82.5%, p < 0.001). This difference may be because surgical antibiotic use, particularly for prophylaxis, is generally more protocol-driven, a point also emphasized by Bratzler DW et al. [19]. In contrast, physicians in medical specialties often deal with diagnostically uncertain or polymicrobial infections, which may lead to the use of broader-spectrum agents or multiple antibiotic prescriptions. This is reflected in the higher average number of antibiotics per patient in medical units (1.43 ± 0.6 vs 1.18 ± 0.5, p < 0.001), a finding also noted by Klein EY et al. [20].

It was noted that patients admitted to surgical specialties were more likely to stay in the hospital for longer than 7 days than those admitted to medical specialties (78.8% vs 42.0%, p < 0.001). This difference is probably due to the procedures and recuperation periods involved in surgical care. Many medical admissions, on the other hand, are shorter and do not require procedures.

De-escalation of antibiotics was not consistently implemented, especially in critical care settings, where numerous patients continued on initial broad-spectrum therapy without prompt modification. A comparable trend was noted in General Medicine and Paediatrics, suggesting that the difficulties related to de-escalation may differ across specialties. Tabah A et al. have also noted these challenges [21], especially in critically ill patients, where clinical uncertainty often makes clinicians hesitant to stop therapy.

Overall, our findings are aligned with the goals of India’s National Action Plan on Antimicrobial Resistance, which emphasizes the need for more rational antibiotic use [22]. The differences observed across departments suggest that a one-size-fits-all approach to stewardship may not be effective. Instead, interventions should be tailored to the needs of individual specialties, with particular focus on medical wards and intensive care units. Strategies such as antibiotic time-outs, ongoing audit with feedback, and strengthening de-escalation practices could help improve prescribing patterns. Comparable challenges, especially in critical care settings, have also been reported by Walia K et al. [23].

Limitations 

This study is a single-center study and is cross-sectional in nature; therefore, it reflects prescribing practices at a single point in time without capturing changes over time. In addition, we did not assess clinical outcomes beyond length of hospital stay, which limits our ability to draw conclusions regarding the direct impact of antibiotic use on patient outcomes.

## Conclusions

This study highlights significant gaps in antimicrobial stewardship practices, with notable variation in prescribing patterns across departments. CCM and General Medicine showed greater potential for improvement in adherence to institutional guidelines and optimization of antibiotic use. A key concern was the continued use of Watch-category antibiotics without appropriate review. There is a need to strengthen stewardship through targeted, department-specific interventions, such as timely de-escalation, regular therapy review, and prospective audit and feedback, to promote rational antibiotic use and improve patient outcomes.
